# Communication in fragile X syndrome: Patterns and implications for assessment and intervention

**DOI:** 10.3389/fpsyg.2022.929379

**Published:** 2022-12-22

**Authors:** Anne Hoffmann

**Affiliations:** ^1^Department of Communication Disorders and Sciences, Rush University Medical Center, Chicago, IL, United States; ^2^Department of Pediatrics, Rush University Medical Center, Chicago, IL, United States

**Keywords:** fragile X syndrome, language, communication, intellectual disability, assessment, intervention

## Abstract

Fragile X syndrome (FXS) is the most common cause of inherited intellectual disability and is associated with a high rate of autism diagnosis. Language delays have been noted in the areas of overall communication and the specific areas of receptive, expressive, and pragmatic language, as well as in development of speech sounds and literacy. It has been widely noted that those individuals with a diagnosis of both FXS and autism tend to have more significant intellectual disability and language disorder. In this study, the research exploring the FXS language phenotype is presented, and the roles of cognition, autistic symptomatology, and gender are highlighted as possible. Implications for assessment and intervention approaches based on the strengths and weaknesses of the FXS language phenotype are provided.

## 1. Introduction

### 1.1. Fragile X syndrome

Fragile X syndrome (FXS) is the most common inherited form of intellectual disability with an estimated prevalence of 1/4,000–1/6,000 in males and 1/8,000 in females in the Western world (Coffee et al., 2009). While research in other areas of the world has been historically limited, there is some evidence that prevalence is lower in some Eastern countries, such as China, and higher in some Middle-Eastern countries, such as Egypt (Meguid et al., 2007; Niu et al., 2017). This single-gene disorder stems from the expansion of a trinucleotide sequence (CGG) on the X-chromosome (Willemsen et al., 2011). Once the expansion reaches >200 repeats, it is termed a full mutation and typically the gene becomes methylated, which results in the gene being turned off and production of fragile X messenger ribonucleic protein (FMRP) is reduced or ceased (Kaufmann and Reiss, 1999). FMRP is critical for overall development, and its reduction or absence is the underlying factor in the phenotypic expression of FXS (Casingal et al., 2020). The variance in prevalence between males and females is secondary to the x-linked nature of FXS, as females carry a “protective-X” which may mitigate the effects of the methylated gene (Loesch et al., 2004). The effects of this altered level of FMRP are pervasive, with clinically significant developmental delay, learning disabilities, social and behavioral challenges, anxiety, and executive function deficits being commonly reported (Gallagher and Hallahan, 2012).

Two additional areas frequently associated with FXS are intellectual disability (ID) and autistic characteristics, with increased language delay noted with increased ID and severity of autistic features (Oakes et al., 2013). Studies indicate that the majority of males with FXS will have a moderate to severe ID (Hessl et al., 2009) and 25% of females will have some form of ID (Hagerman et al., 1992). The rate of autism diagnosis is much higher in FXS than in typical development (TD), with approximately 50%–67% of males and 20% of females meeting criteria for autism spectrum disorder (ASD; Wang et al., 2010). This range likely stems from multiple sources, including variance in how ASD is diagnosed (e.g., parent report vs. direct measure of current behavior; standardized assessment vs. clinical judgment). Further, the question of whether the ASD present in FXS is the same ASD found in non-syndromic cases has been the topic of substantial debate (see Abbeduto et al., 2014 for review). The debate has primarily hinged on the observation that those individuals with FXS who also meet criteria for ASD (hereafter referred to as FXS + ASD) have lower intelligence quotient (IQ) on average than those who do not meet criteria (referred to as FXS-O; Bailey et al., 2001; Kaufmann et al., 2004; Lewis et al., 2006), which asks the question of whether FXS + ASD simply represents the more affected end of the spectrum of FXS phenotypic presentation. While that question is beyond the scope of this paper, in an effort to clarify research findings, we will highlight those studies that have compared FXS-O and FXS + ASD when such distinctions are possible.

For this review, we consider several areas in communication and language development. Communication refers to the broad concept of how an individual relays and receives messages with others, including the prelinguistic communication associated with very early development. This is frequently included in measures of adaptive behavior and the mode of communication can vary (e.g., gestures, use of a speech-generating device, spoken messages). As multiple studies have used communication in its broadest sense to assess if individuals possessed this capacity, we have included it as a separate category, in addition to language. Language is a form of communication that utilizes a specific set of symbols mutually understood by the creator and receiver of the messages (Gumperz, 1967) and for this review, we use this to refer to spoken language. Within language, we discuss receptive language (what is understood), expressive language (how an individual communicates), and pragmatic language (how communication is used in social contexts). Within receptive and expressive language, we examine overall patterns as well as the separate areas of morphology and syntax (i.e., morphosyntax/grammar) and vocabulary as permitted by the research that has been done in these areas. We also review current findings for speech sound and literacy development. Comparisons with other groups, most commonly Down syndrome (DS) and idiopathic ASD, will be highlighted to demonstrate phenotypic-specific tendencies in communication. The roles of gender, cognition, and autistic symptomatology in the communication profile are considered as possible. For interpretation of findings, infants refer to children 1 year of age and younger, very young children refer to those individuals ages 1 to 3 years, children (i.e., boys and girls) to those individuals aged 4–11 years, adolescents to those individuals aged 12–17, and adults (i.e., men and women) to those individuals 18 years and older. For overarching trends across the lifespan, the terms males and females are used. We also use the terms boys/men/males and girls/women/females to refer to biological sex as determined at birth. Finally, implications of the FXS language phenotype for clinical assessment and intervention are considered.

## 2. Materials and methods

**Figure 1:**
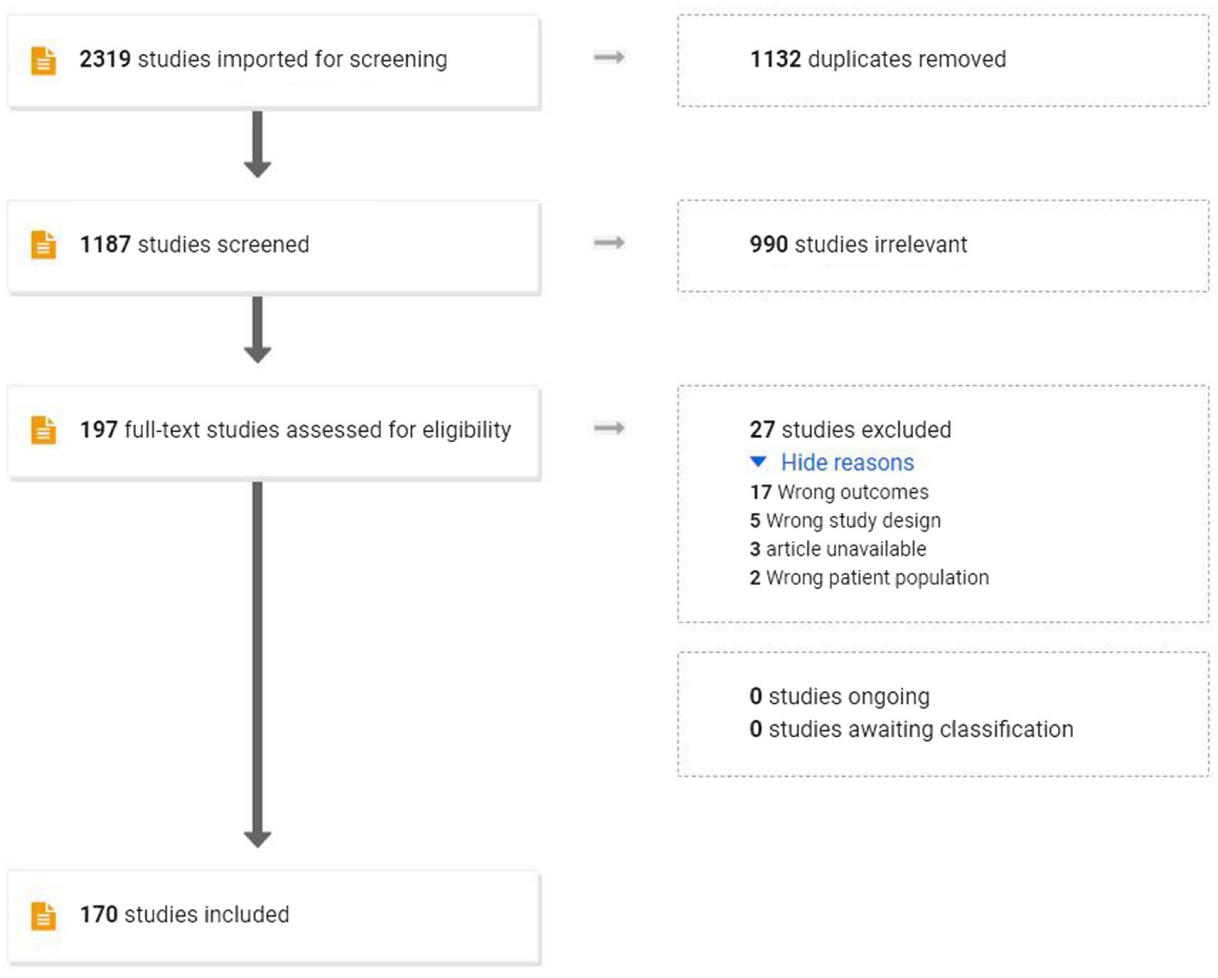
PRISMA diagram.

For the current study, a comprehensive literature search was developed and run by an experienced medical librarian in October 2022 in the following databases: PubMed/MEDLINE, Scopus, CINAHL, PsycINFO, ComDisDom, the Cochrane Database of Controlled Trials, and the Cochrane Database of Systematic Reviews. Google Scholar was searched as well. Both controlled vocabularies (e.g., MeSH terms) and keywords in the title or abstract fields were searched. There were no restrictions on geography or age of participants. Animal studies were excluded. Additionally, a hand search was conducted of the reference lists of selected articles. A reproducible search strategy is attached, see Appendix 1. This initial search resulted in 2319 studies being imported for screening, of which 1,132 duplicates were removed, leaving 1,187 studies to be screened using title and abstract. These were screened and 990 were excluded secondary to one of the following criteria (1) no language outcomes; (2) participants did not include individuals with FXS; (3) was not a peer-reviewed study (e.g., book chapter, dissertation); and (4) article was not available in English. This resulted in 197 studies being assessed *via* full-text review. Twenty-seven of these studies were excluded for the following reasons: outcomes (17, outcomes did not include separate communication measures); study design (5, only case studies were provided); patient population (2, full mutation FXS was not included or details regarding the FXS performance were not provided); and Article was unavailable (3). Of the remaining 170 articles, 5 were review articles and 55 were published prior to 2009, which was the date of the latest comprehensive review. As such, the focus of this paper will be on research found within the remaining 110 studies, with comparisons drawn to the findings of previous reviews. The PRISMA diagram can be seen in Figure 1.

## 3. Results and discussion

### 3.1. Communication

As noted in previous reviews (Abbeduto and Hagerman, 1997; Abbeduto et al., 2007; Finestack et al., 2009), individuals with FXS evidence communication delays early in development. Measurable delays have been noted as young as 6 months for males with FXS (Wheeler et al., 2021). This results in many individuals with FXS remaining as prelinguistic communicators far later than what is seen in typical development (Brady et al., 2006). These delays extend beyond the milestones of spoken language; the areas of communicative gestures, eye gaze, vocalizations, and communicative functions have all been shown as delayed relative to typical development (Flenthrope and Brady, 2010; Hinton et al., 2013; Marschik et al., 2014; Kover et al., 2015; Hahn et al., 2017; Rague et al., 2018; Hughes et al., 2019; Mattie and Hamrick, 2022).

Despite these delays, there is clear evidence that individuals with FXS do progress in their communicative ability (Bailey et al., 2009; Wheeler et al., 2021). Bailey et al. (2009) performed a study in which a survey was distributed to a large sample of individuals with FXS and their caregivers to assess adaptive behavior, including communication. Participants ranged in age from 1 to 62 years. Results indicated that the majority of adult males and females with FXS had reached functional communication levels by adulthood (i.e., single words or signs) and most females had reached advanced communication levels (i.e., complex sentences and conversations). Of note, there were increased percentages of individuals in each age group demonstrating the various communication skills (e.g., single words, signs, complex speech), indicating that skills continued to develop, albeit at a slower pace than TD. Because growth in FXS is slower than in TD, standard scores will sometimes show a decline (Klaiman et al., 2014). However, it is important to note that the decline in standard scores does not necessarily mean a loss of skill. Rather, as has been demonstrated in performance on cognitive assessments, the rate of skill acquisition in FXS often does not show the rapid acceleration of growth found in typical development, which increases the gap between FXS and neurotypical individuals (Hall et al., 2008).

#### 3.1.1. Related factors

The role of gender in communicative development has demonstrated the expected strengths in females relative to males with FXS although females with FXS may still show delays relative to TD (Caravella and Roberts, 2017; Wheeler et al., 2021). Females with FXS often manifest delays by the age of 12 months, with a rate of growth that while faster than males with FXS is still slower than TD (Caravella and Roberts, 2017; Wheeler et al., 2021). However, studies have noted significant variability in communicative performance in females, so higher or lower performance is possible (Bailey et al., 2009; Flenthrope and Brady, 2010; Klaiman et al., 2014; Wheeler et al., 2021).

Some research has focused on cognitive processes that might underlie the communication delay found in FXS. There is evidence that very young children with FXS had atypical face-scanning patterns, suggesting differences in visual attention (D'Souza et al., 2015, 2020). In general, nonverbal cognition has strong relationships with communication ability across the lifespan, with lower nonverbal ability being correlated to decreased communication (Reisinger et al., 2019). However, in general, it appears that early communication is roughly commensurate with other developmental areas (Reisinger et al., 2019). Other studies have suggested that variance in parental input could impact communication development, as increased maternal responsivity is associated with steeper trajectories of growth (Warren et al., 2017).

Recent research has found that increased autistic symptomatology is generally associated with greater communication delay in FXS, and this is in agreement with previous research (Abbeduto and Hagerman, 1997; Abbeduto et al., 2007; Finestack et al., 2009; Fielding-Gebhardt and Warren, 2019; Mattie and Hamrick, 2022). The impact of autistic symptomatology has been demonstrated in gestures, gaze shift, and initiation of joint attention (Flenthrope and Brady, 2010; Hahn et al., 2016, 2017; Brewe et al., 2018; Rague et al., 2018; Hughes et al., 2019). However, the majority of these studies also found strong correlations between these same areas and nonverbal cognition, which speaks toward the difficulty in separating these two characteristics in the FXS phenotype, a challenge that has been discussed at length (Abbeduto et al., 2014).

### 3.2. Receptive language

As noted in previous reviews, receptive language is delayed with impairments evidence in comprehension of vocabulary and morphosyntactic structures (Abbeduto and Hagerman, 1997; Abbeduto et al., 2007; Finestack et al., 2009). The review by Finestack and Abbeduto (2010) presented mixed study results when comparing receptive language in FXS to children with TD matched on nonverbal cognitive development. Some studies have found that receptive language in FXS was on par with controls matched on nonverbal cognition (Abbeduto et al., 2003; Roberts et al., 2007) while others show the FXS group falling below (Roberts et al., 2001; Price et al., 2007). Recent studies have examined specific receptive language domains, although as highlighted below, there is still debate.

#### 3.2.1. Receptive vocabulary

When examining specific areas of receptive language in individuals with FXS, vocabulary has appeared as a relative strength, with skills in this area outpacing syntax and sometimes nonverbal cognition in adolescents and adults (Thurman et al., 2017b; Hoffmann et al., 2019). Receptive vocabulary increases with age (Brady et al., 2020), and its position as a relative strength has been shown across development (Thurman et al., 2017b). When comparing receptive vocabulary in FXS to what is found in other neurodevelopmental diagnoses, there have been mixed findings. Some studies have found that children and adolescents with FXS have stronger receptive vocabulary skills than individuals with DS or ASD matched on nonverbal cognition (Thurman et al., 2017b; Del Hoyo Soriano et al., 2018; Thurman and Hoyos Alvarez, 2020). Others find no difference between the groups (Finestack et al., 2013).

#### 3.2.2. Receptive morphosyntax

Comprehension of grammar has been shown as commensurate with nonverbal cognition in some studies (Thurman and Hoyos Alvarez, 2020) and below nonverbal cognition in others (Oakes et al., 2013). It is possible that there are certain contexts which impact receptive morphosyntax. Oakes et al. (2013) propose that comprehension of sentences with a high demand of auditory sequencing or ones that lack lexical supports might be more problematic for individuals with FXS. When compared to peers with TD matched on nonverbal cognition, male children and adolescents with FXS still tend to fall below on receptive morphosyntax measures (Finestack et al., 2013; Oakes et al., 2013; Martin et al., 2013b), but there are studies that show similar performance (Finestack and Abbeduto, 2010). Comparisons with other groups have found equivalent skills between FXS and both ASD and DS when individuals are matched on nonverbal IQ (NVIQ; Finestack et al., 2013; Thurman et al., 2017b).

While the mixed results make a summary difficult, there are clearly delays relative to chronological age. The variance in study results is likely to stem from methodological differences. For example, the age of participants, the inclusion/exclusion of females, how language and cognition were assessed, whether age equivalent scores were used, these could all impact how groups compare against each other.

#### 3.2.3. Related factors

Studies specifically examining receptive language in females with FXS have found the expected trend of more preserved abilities as compared to males, although many of the participants still fall below chronological age expectations (Roberts et al., 2007; Sterling and Abbeduto, 2012; Joga-Elvira et al., 2021). Sterling and Abbeduto (2012) found that similar to males with FXS, females also had receptive vocabulary skills that were generally higher than their nonverbal cognitive ability, although there was considerable variation across participants. Receptive syntax is generally weaker than receptive vocabulary, just as was described in males with FXS (Oakes et al., 2013). While studies specifically examining receptive language in females with FXS are limited, several have included females within the participant group. Many of these studies also found that while the females had stronger language skills overall, they had similar relationships between receptive language and other traits (e.g., autistic symptomatology, nonverbal cognition) as males with FXS (Finestack et al., 2013; Oakes et al., 2013; Hoffmann et al., 2019). However, Brady et al. (2020) and Pierpont et al. (2011) both found a steeper trajectory for some receptive language skills in female children and adolescents with FXS as compared to males.

Across studies, nonverbal cognition has been demonstrated as an important factor for receptive language. Brady et al. (2020) found that NVIQ, as well as parenting style, was related to growth in receptive and expressive vocabulary over time. Pierpont et al. (2011) examined specific cognitive areas, with phonological memory and working memory being strongly correlated to receptive vocabulary and syntax in boys with FXS, while in girls, overall cognition was strongly correlated but not those specific subdomains.

The role of autism in receptive language is closely related to cognition. Thurman and Hoyos Alvarez (2020) and Thurman et al. (2017b) found that autistic symptomatology and nonverbal cognition predicted receptive vocabulary in boys with FXS regardless of ASD status. Interestingly, the type of autistic symptomatology was important, with severity in restricted and repetitive behaviors having strong correlations to delays in receptive vocabulary and other language areas for children (Thurman and Hoyos Alvarez, 2020). In adolescents and adult with FXS, recent studies have not shown a difference in receptive language based on autism status once analyses are adjusted for nonverbal cognition (McDuffie et al., 2012; Hoffmann et al., 2019). However, when autistic symptomatology was assessed as a continuous metric, it was a significant predictor of receptive vocabulary and grammar (McDuffie et al., 2012). This suggests that the relationship between autistic behaviors in FXS may benefit from a more nuanced assessment than a simple categorical approach.

### 3.3. Expressive language

As in receptive language, there is general consensus that expressive language in FXS is significantly delayed relative to chronological age expectations (Abbeduto and Hagerman, 1997; Abbeduto et al., 2007; Finestack et al., 2009). These delays have been found in previous studies in both expressive vocabulary and expressive morphosyntax, when assessed through traditional standardized assessment as well as language sampling. Previous reviews have described the expressive language ability of males with FXS as falling below that of children with TD matched on cognition (Finestack et al., 2009), but more mixed findings are reported in vocabulary and morphosyntax (Abbeduto and Hagerman, 1997; Abbeduto et al., 2007).

#### 3.3.1. Expressive vocabulary

Recent studies have shown expressive vocabulary in boys with FXS as impaired relative to TD children matched on nonverbal mental age (Kover et al., 2012; Martin et al., 2013b). Longitudinal studies using standardized measures have found increases in vocabulary in childhood and adolescence, although there may be a decrease in rate of growth during late childhood (Martin et al., 2013b; Brady et al., 2020). When lexical diversity—a measure of expressive vocabulary—has been calculated from language samples, there seems to be a decrease in number of different words used by adolescent males in conversation, despite an increase in the talkativeness (Del Hoyo Soriano et al., 2020).

Comparisons have been made between individuals with FXS and those with ASD and DS, matched on either nonverbal cognition, mean length utterance (MLU), and or autistic symptomatology. Individuals with FXS have generally had stronger performance on expressive vocabulary measures than individuals with DS matched on nonverbal cognition (Finestack et al., 2013; Martin et al., 2013b). FXS as compared to ASD has not evidenced differences in lexical diversity when NVIQ was used for matching (Kover et al., 2012), but when MLU and autistic symptomatology were used the FXS group had fewer different words than the ASD group (Hilvert et al., 2020). However, the participants with FXS in Hilvert et al.’s study had much lower scores on a standardized assessment of vocabulary and NVIQ than the group with ASD which could impact their performance.

#### 3.3.2. Expressive morphosyntax

Expressive morphosyntax is also below what is seen in TD when nonverbal cognition is controlled (Estigarribia et al., 2012), and there is also evidence that boys with FXS have more impairment in expressive grammar as compared to expressive vocabulary (Martin et al., 2013b). When specific grammatical forms are examined, individuals with FXS seem to follow an atypical developmental pattern. While children with FXS fall below children with TD matched on nonverbal mental age in general measures of expressive grammar and MLU, they acquire some later developing forms (e.g., third-person singular markers) earlier than would be predicted by MLU (Estigarribia et al., 2011; Sterling et al., 2012; Komesidou et al., 2017).

Compared to groups with developmental language disorder (DLD) and TD matched on MLU, boys with FXS performed better on certain morphological structures such as finiteness marking than the group with DLD and even out-performed the group with TD on third-person singular forms (Haebig et al., 2016). This could indicate that in FXS, MLU does not have the same relationship to specific morphological forms that is seen in TD (Rice and Wexler, 2001; Rice et al., 2010; Haebig et al., 2016). When compared to individuals with DS, frequently noted as having relative weakness in expressive language skills, individuals with FXS have mostly been found as having stronger expressive morphosyntax (Martin et al., 2009; Finestack and Abbeduto, 2010; Finestack et al., 2013; Martin et al., 2013a).

Longitudinally, Martin et al. found that the boys with FXS did make gains over time on standardized assessments of expressive morphosyntax, but the rate of growth was slower than what is seen in TD, similar to what was seen in the group with DS. This slower growth has been replicated in other studies, and the possibility of a plateau in skill development has been noted (Komesidou et al., 2017). When adolescents with FXS were assessed over time using language samples, there was a decrease in syntactic complexity despite an increase in the overall amount of utterances (Del Hoyo Soriano et al., 2020). These could reflect a discrepancy between growth in standardized assessment as compared to functional use of structural language, as was seen in expressive vocabulary.

#### 3.3.3. Related factors

Early expressive language delays occur in both males and females with FXS, although as in other areas, females tend to be more mildly affected (Brady et al., 2006). Research specifically comparing males and females with FXS in expressive language has found the expected trends of stronger performance and growth in females, with considerable individual variability (Finestack and Abbeduto, 2010; Komesidou et al., 2017). Some research indicates that female children and adolescents with FXS have MLU within the age expectations, and that NVIQ is not predictive of this ability (Sterling and Abbeduto, 2012). Others have found that NVIQ is predictive of either MLU (Komesidou et al., 2017) or complex syntax (Kover and Abbeduto, 2019). Given the tendency of males with FXS to have complex syntax above what their MLU would predict, this is an area that merits further research.

Several studies have found that expressive language ability and growth is predicted by nonverbal cognitive skills (Price et al., 2008; Pierpont et al., 2011; Estigarribia et al., 2012; Martin et al., 2013b; Komesidou et al., 2017). As in receptive language, phonological and working memory appear correlated with expressive vocabulary and syntax (Pierpont et al., 2011; Estigarribia et al., 2012; Kover and Abbeduto, 2019).

There is evidence that autistic symptomatology is linked to increased expressive language deficits across development. A study examining parent-reported early milestones found an average delay in first words of 3 months for very young boys with FXS-O and 13 months for FXS + ASD (Hinton et al., 2013). However, a study that examined early gesture usage did not find a correlation between autistic symptomatology and gestural delay once nonverbal ability was added as covariate (Rague et al., 2018). Haebig and Sterling (2017) compared receptive-expressive vocabulary profiles in adolescents with FXS + ASD and ASD. They found that despite having similar profiles of autistic symptomatology, the groups differed significantly in their vocabulary profiles, with the participants with ASD having a high rate of gaps in receptive-expressive vocabulary skills that favored expressive vocabulary and participants with FXS + ASD having a much lower rate (Haebig and Sterling, 2017). In addition, there is some evidence that boys with FXS + ASD show atypical acquisition of grammatical morphemes in a manner more similar to what is seen in ASD, although this has not included a comparison to boys with FXS-O (Sterling, 2018). Studies examining syntax in boys with FXS-O and FXS + ASD have not consistently found differences between the two once NVIQ is considered (Roberts et al., 2007; Kover and Abbeduto, 2010; Estigarribia et al., 2012.

### 3.4. Pragmatic language

Pragmatic language refers to the use of communication in social contexts, including communicative exchanges, production of contingent and appropriate messages, understanding varying points of view, etc. (American Speech-Language-Hearing Association, 2022). This is a core deficit in ASD and given the high rate of ASD diagnosis in FXS, it is unsurprising that this is a frequent area of weakness. In previous reviews, FXS has been noted as having difficulty in initiating and maintaining discourse, repairing communication breakdowns, and creating narratives. Increased rates of pragmatic deficits are also noted in populations with intellectual disability, language disorder, attention deficits, and other neurodevelopmental disorders (Tager-Flusberg, 2004; Towbin et al., 2005; Hoffmann et al., 2013; Smith et al., 2017; Diez-Itza et al., 2022). Pragmatic expectations are derived from cultural expectations (Hyter, 2007), creating some level of variance in terms of what is considered typical, but there are common patterns that emerge in FXS regardless of culture.

Aside from the linguistic characteristics that will be discussed, there are non-spoken elements to pragmatic language that are atypical in the FXS phenotype. Eye gaze aversion has been extensively noted as occurring regardless of the presence of other autistic symptomatology (see Hagerman and Hagerman, 2002 for review) and in both males and females, although females do continue to show increased variability in presentation (Hessl et al., 2006; Hall et al., 2009). Other nonverbal areas that are reported as being atypical in boys with FXS are intonation, gesture use, and facial expression (Klusek et al., 2014). When comparing FXS to ASD, there is mixed evidence. Some studies found that boys with FXS + ASD perform similarly to boys with ASD matched on chronological age and language ability (Losh et al., 2012; Klusek et al., 2014). Other research has found that individuals with FXS + ASD have some key differences in core autistic traits when compared to ASD (Wolff et al., 2012; McDuffie et al., 2015; Lee et al., 2016; Thurman et al., 2017b). A study by McDuffie et al. (2015) found that boys with FXS + ASD matched to a group of boys with ASD on both chronological age and autistic symptomatology had different patterns of symptoms. The group with FXS + ASD manifested less impairment in social smiling, range of facial expressions, gesture use, and restricted interests than the group with ASD. There is also evidence that social responsivity is less impaired in FXS + ASD than ASD (Wolff et al., 2012; Thurman et al., 2017b; Hong et al., 2019).

Assessments of meta-pragmatics (i.e., the understanding of what *should* occur) have found that males with FXS perform similarly to individuals with other forms of ID (e.g., DS; Losh et al., 2012; Klusek et al., 2014), and higher than individuals with ASD (Losh et al., 2012). However, functional use of those same skills, as measured by caregiver report, reveals similar performance between boys with FXS and ASD (Losh et al., 2012) and weaker performance than boys with DS (Del Hoyo Soriano et al., 2018). This suggests that the manifestation of pragmatic deficits during interactions is not reflective solely of intellectual disability.

Narrative ability (i.e., story-telling) is a key element of social interaction. In FXS, there is demonstrated impairment in narrative processing and creation (Estigarribia et al., 2011). However, in some specific areas (e.g., inferential language and providing introductory details), children and adolescents with FXS perform at similar or higher levels as TD children matched on nonverbal cognition (Finestack et al., 2012; Hogan-Brown et al., 2013). Comparisons to other groups have shown no difference in narrative macrostructure for boys with FXS and individuals with DS, ASD, and TD matched on either language or cognition (Finestack et al., 2012; Hogan-Brown et al., 2013).

Conversational analyses have revealed that males with FXS produce significantly more non-contingent remarks (i.e., responses that are tangential to the preceding remark) than males with TD who are matched on language ability (Wolf-Schein et al., 1987; Sudhalter and Belser, 2001; Martin et al., 2013b) as well as reduced usage of conversational repair strategies (Abbeduto et al., 2008; Barstein et al., 2018). Another key finding noted consistently in language analyses of FXS is excessive self-repetition of certain phrases and topics, also termed perseveration (Losh et al., 2012; Martin et al., 2012, 2013b, 2018; Del Hoyo Soriano et al., 2018; Friedman et al., 2018; Diez-Itza et al., 2022). This repetition is found in several forms, including immediate repetition of a specific word or phrase (e.g., “She’s gonna be a statue, gonna be a statue”), repetition of a specific conversational device that does not add information to the conversation (e.g., “Right on”), or repeatedly returning to a specific topic of conversation (Murphy and Abbeduto, 2007). There is evidence that this is a key phenotypic element to FXS, as it is found regardless of non-verbal cognitive or language ability and in both males and females with FXS (Martin et al., 2018; Hoffmann et al., 2022). Interestingly, levels of self-repetition have distinguished groups with FXS and ASD, with FXS showing higher levels of topic and phrase repetition and ASD showing higher rates of conversational device repetition (Hilvert et al., 2020; Hoffmann et al., 2022).

#### 3.4.1. Related factors

As in other areas, females with FXS frequently demonstrate less severe pragmatic impairment than males, although there is considerable variability (Abbeduto et al., 2008; Thurman et al., 2017a; Martin et al., 2020; Neal et al., 2022). Girls show deficits in signaling of non-comprehension as compared to TD peers matched on cognition, and there has been some research showing decreased responsivity in girls with FXS as they reach adolescence when asked to repair a communication breakdown (Thurman et al., 2017a; Martin et al., 2020). Females with FXS who also meet criteria for ASD have been shown to be less likely to signal non-comprehension, initiate conversation, or make contingent remarks in conversation than those with FXS-O or individuals with DS and TD matched on nonverbal cognition.

In infants with FXS, lower NVIQ has been shown as related to reduced initiation of joint attention (Brewe et al., 2018). Nonverbal cognition was correlated to overall ASD severity and predictive of the level of restricted and repetitive behaviors (RRBs; Abbeduto et al., 2020). However, in Haebig et al. (2020), NVIQ did not account for different performance on measures of autistic symptomatology. Similarly, a study examining question usage in boys with FXS + ASD did not find NVIQ correlated to the rate of inappropriate questions, personal questions, or requests for clarification (Friedman et al., 2020).

Some studies have found evidence of group differences based on ASD diagnosis, with boys with FXS + ASD demonstrating more impairment in pragmatic understanding and skills than FXS-O even after controlling for nonverbal cognition (Losh et al., 2012; Martin et al., 2013b; Klusek et al., 2014). The pattern of autistic symptomatology seems to vary with age. McDuffie et al. (2015) found that increased rates of RRBs were the determining factor for a comorbid diagnosis of ASD for children and adolescent males with FXS. However, when male adolescents and young adults were assessed for autistic traits, there were few RRBs with the exception of stereotyped and idiosyncratic language and more impairment in the social affective domain (Abbeduto et al., 2019).

### 3.5. Speech

A review of speech sound development by Barnes et al. (2006) describes a pattern of reduced intelligibility in FXS as compared to TD. Formal assessments of articulation found that boys with FXS have error patterns similar to nonverbal mental-age-matched boys with TD on single-word tasks (Paul et al., 1984; Roberts et al., 2005) and that there are increased errors on multisyllabic words as compared to single syllable words with significant effects for both nonverbal cognition and chronological age (Barnes, 2006).

Recent studies have reflected these same findings, with on-going evidence of articulation deficits as well as atypical rate of speech (Madison et al., 1986; Sudhalter et al., 1990; Ferrier et al., 1991; Belser and Sudhalter, 2001). Intelligibility in connected speech is lower than what would be predicted by performance on single words for males (Barnes, 2006; Barnes et al., 2006). This is evidenced by similar performance to boys with TD matched on nonverbal cognition on single-word tasks, but significantly lower performance on measures assessing intelligibility in connected speech (Barnes et al., 2006). Boys with FXS have also shown lower intelligibility in connected speech than boys with ASD matched on autism severity (Hilvert et al., 2020). Compared to boys with DS matched on nonverbal cognition, boys with FXS typically have better performance on all speech-sound and intelligibility tasks (Barnes et al., 2009; Kover et al., 2012; Martin et al., 2018). Acoustical analyses of speech samples have also revealed that the perceived rapid rate of speaking may stem from fewer pauses between words instead of faster rate of articulation (Zajac et al., 2006). There is also evidence that up to 50% of young adult males with FXS meet criteria for cluttering, a fluency disorder that is associated with irregular rate of speech and decreased intelligibility, with the unexpected finding that nonverbal cognition was positively correlated with increased risk of cluttering (Bangert et al., 2022).

#### 3.5.1. Related factors

At this time, we are unable to find any published studies examining speech sound patterns in females with FXS.

Nonverbal cognition has shown strong relations to intelligibility, with lower NVIQ being associated with lower intelligibility (Barnes et al., 2006; Shaffer et al., 2020). Similarly, individuals with FXS + ASD have shown a tendency to have decreased intelligibility compared to FXS-O (Barnes et al., 2009; Estigarribia et al., 2011; Kover et al., 2012; Klusek et al., 2014; Shaffer et al., 2020), but there have been exceptions (Barnes et al., 2009; Estigarribia et al., 2011).

### 3.6. Literacy

Limited research exists regarding literacy development in FXS, as such this discussion will not separate out related factors. A large national survey of families living with individuals with FXS revealed that only 44% of adult males were able to read basic picture books and just 59% knew letter sounds (Bailey et al., 2009). A study comparing boys with FXS to boys with TD matched on nonverbal cognition found that boys with FXS had similar or superior performance on word reading and passage comprehension (Klusek et al., 2014). However, this same study found that phonological awareness was lower in the boys with FXS as compared to boys with TD, and that this skill was significantly correlated with autistic symptomatology (Klusek et al., 2014). A follow-up study for these same participants demonstrated that the boys with FXS acquired phonological awareness at a similar rate to the boys with TD once nonverbal cognition was controlled, although both this study and others have found a plateau in phonological awareness growth for boys with FXS at around the age of 10 years (Roberts et al., 2005; Bailey et al., 2009; Adlof et al., 2015).

Despite the relative strength found in early word recognition, there is general consensus that phonological awareness is an important predictor of reading ability, just as in typical development (Roberts et al., 2005; Bailey et al., 2009; Adlof et al., 2015). Research with adolescent boys with FXS has strengthened that understanding as phonological awareness skills had a strong positive relationship with oral word reading ability (Adlof et al., 2018).

## 4. Clinical implications

### 4.1. Assessment

Standardized language and educational assessments of individuals with FXS are central to the creation of an appropriate intervention plan (Salvia et al., 2016). Unfortunately, given their global language delays, there are frequently limited options for norm-referenced standardized assessments that have items for the appropriate skills (Hoffmann et al., 2020). As an older individual with FXS may still be at an early developmental language level, e.g., an adult who is at the two-word phrase level, an assessment that expects fluent, multi-word utterances would be inappropriate. This is especially true for the areas of syntax and morphology, which as discussed above can be specific areas of weakness. Clinicians are often faced with the choice of using an assessment that is appropriate for an individual’s chronological age or using one that is appropriate for their language level. Hoffmann et al. (2020) found that the majority of individuals with FXS across a wide-age range were able to complete a standardized assessment meant for their chronological age, but that a significant percentage did not achieve a valid score (i.e., they received a score at the floor of the assessment, which does not reflect language variability).

This lack of appropriate measures often forces the use of instruments outside of their intended age range, which creates the difficulty of what scores to report. While age-equivalency scores are still frequently seen in both research and clinical reports, they are concerning psychometrically as they do not represent an equal interval scale (Salvia et al., 2007). This lack of appropriate measures has been cited as a leading cause of the failure of several clinical trials in FXS (Berry-Kravis et al., 2013b; Budimirovic et al., 2017), besides limiting the ability of clinicians to accurately assess their clients.

One option that can be considered is caregiver report, these are frequently used as they can provide information about behaviors across contexts as well as skills that are difficult to elicit in clinical or educational settings. Three commonly used caregiver report measures have been adapted for the specific profiles found in FXS, and are used to assess maladaptive behavior (Kerr et al., 2014) and social-communication/responsivity (Kidd et al., 2014). However, caregiver reports need to be combined with objective measures to gain an accurate picture of functioning (Bishop and McDonald, 2009).

Another choice that allows for an objective measure of expressive language across a wide range of language abilities is communication or language sampling. For individuals relying on primarily non-speaking means of communication (e.g., triadic eye gaze, gestures), communication sampling can allow for assessment of those often subtle behaviors (Brady et al., 2012; Hahn et al., 2017). These have been shown as effective in a wide range of populations and ages, including FXS (Brady et al., 2012; Hahn et al., 2017). For individuals regularly using two-to-three-word phrases, an expressive language sampling (ELS) protocol has been developed and shows strong psychometrics in its use in FXS (Berry-Kravis et al., 2013a; Abbeduto et al., 2020; Shaffer et al., 2020). It has been shown to differentiate between diagnoses, and to be able to characterize syntax, vocabulary, and pragmatics in FXS and other populations with varying levels of language ability (Abbeduto et al., 2020; Shaffer et al., 2020; Hoffmann et al., 2022).

Given these findings, clinicians will need to rely on a combination of clinical reasoning and research-based recommendations. What is clear is that assessment of individuals with FXS will likely require a clinician to think outside of the traditional norm-referenced standardized assessments. In order to gain an accurate understanding of ability, it is likely that multiple types of assessment will need to be used.

### 4.2. Intervention

Most individuals with FXS will receive services early in life, with declining rates of service utilization as they age (Martin et al., 2013a). There is growing research indicating that increased caregiver responsivity with young children is highly predictive of later language ability in FXS (Brady et al., 2014, 2020). There has also been some research as to how a parent-mediated intervention can increase social responsivity in children with FXS (Alfieri et al., 2021). This means that caregivers should be actively involved in treatment and clinicians should pay particular attention to fostering more responsive interactions. This includes supporting the use of augmentative and alternative communication (AAC) in the home, which caregivers report as being a useful tool for addressing complex communication needs in FXS (Schladant and Dowling, 2020).

As children become older, caregivers are still an important tool for improving language as there is evidence that caregiver responsivity practices can remain effective later in development with some adjustments (e.g., more commenting and fewer questions; Brady et al., 2020). Shared book-reading has also been shown as an effective tool for increasing the likelihood of sustained verbal interactions between school-aged children with FXS and their caregivers (McDuffie et al., 2016a, 2018; Nelson et al., 2018). The caregivers increased their use of language facilitation strategies (e.g., intonation prompts, modeling of story-related grammar and vocabulary) and the children showed gains in vocabulary and inferential language. The benefit to incorporating a book into this intervention is that it also continues to build on the print awareness and narrative structure needed for literacy (Justice et al., 2009). These practices that have focused on educating caregivers in communication techniques have also been proven effective when delivered *via* telehealth, opening up additional possibilities for families who may have trouble finding a provider familiar with them nearby (McDuffie et al., 2016a,b, 2018; Bullard et al., 2017; Abbeduto et al., 2020; Shaffer et al., 2020; Hoffmann et al., 2022).

Given the growing evidence that reading skills in FXS follow the same path as in TD, i.e., phonological awareness leading to increased oral word reading ability, clinicians should consider how to effectively target this area. Whereas earlier recommendations focused only on whole word recognition (Braden, 2002) secondary to concerns about weaknesses in sequential processing (Hodapp et al., 1992), there is now evidence that individuals with FXS may benefit from the traditional phonics-based approach (Adlof et al., 2018). Adlof et al. (2018) examined whether a widely available computer-based phonics program would be appropriate for a group of adolescent and young adults with FXS. They found that most of the participants (which included both males and females) were able to access and use the intervention which had been developed for use in general education.

These findings provide guidance to clinicians, although future studies examining how to support higher level language skills and school-based practices are still needed. Currently, it appears that embedding language learning opportunities in interactions that happen frequently and consistently are key elements to early language development, similar to what is recommended for other populations with language delay (Snyder et al., 2015). Similarly, growing research indicates that the key elements needed for literacy in the general populations are the same ones needed for individuals with FXS, and they can be supported by already available techniques. While it is likely that clinicians will need to modify to accommodate the FXS phenotype (e.g., providing increased repetition, structuring activities around breaks to decrease anxiety), it is also important to note that it appears that commonly used and recommended approaches to intervention are effective.

## 5. Conclusion

Language in FXS has benefitted from extensive research, highlighting its unique pattern of strengths and weaknesses. In general, individuals with FXS have stronger receptive than expressive language skills, and this tendency begins early in development. In both receptive and expressive language, vocabulary is often an area of strength, as compared to morphology and syntax, and at times exceeds what is expected given nonverbal cognitive abilities. Pragmatics are an area of weakness, although the role that autism comorbidity plays is still a question. Repetitive language appears to be a key component of the FXS phenotype, and its presence is independent of both IQ and autism status. The importance of considering cognition when analyzing language trends is clear, a common theme throughout the research is that when NVIQ is considered, many of the differences between FXS-O and FXS + ASD do not remain. Speech intelligibility is also an area of concern, with correlations to nonverbal cognition. Finally, literacy is an area that has received little attention, despite reports that individuals with FXS have extremely limited literacy skills.

Despite the well-established understanding of language abilities in this population, it is vital that future studies continue to extend assessment and intervention approaches to this population. While the benefits of caregiver responsivity have been made clear, there is scant research on other methods of supporting communication in individuals with FXS, especially once they reach school-age or above, despite clear evidence that they have significant needs. These areas must be addressed if we are to provide the necessary tools for best outcomes over the long-term, and likely includes how to afford caregivers with the required supports over the lifespan.

## Author contributions

AH contributed to the writing of initial draft, subsequent edits, and final preparation of the manuscript.

## Funding

Rush University, College of Health Sciences, provides funds for open-access publication fees.

## Conflict of interest

The author declares that the research was conducted in the absence of any commercial or financial relationships that could be construed as a potential conflict of interest.

## Publisher’s note

All claims expressed in this article are solely those of the authors and do not necessarily represent those of their affiliated organizations, or those of the publisher, the editors and the reviewers. Any product that may be evaluated in this article, or claim that may be made by its manufacturer, is not guaranteed or endorsed by the publisher.

## Appendix 1

Search terms for systematic review.

**PubMed: 743.**

(“fragile X”[tiab] OR “Fragile X Syndrome”[Mesh])

AND (Language[tiab] OR communicat*[tiab] OR conversation[tiab] OR language[tiab] OR linguistic*[tiab] OR literacy[tiab] OR literate[tiab] OR narration[tiab] OR non-verbal[tiab] OR speak[tiab] OR speech[tiab] OR talk*[tiab] OR verbal*[tiab] OR “Language”[Mesh] OR “Literacy”[Mesh] OR “Speech”[Mesh] OR “Narration”[Mesh] OR “Nonverbal Communication”[Mesh]).

NOT (mice OR (animals[mesh] NOT humans[mesh])).

**Scopus: 623.**

(TITLE-ABS (“fragile X”))

AND (TITLE-ABS (language OR communicat* OR conversation OR language OR linguistic* OR literacy OR literate OR narration OR non-verbal OR speak OR speech OR talk* OR verbal*)).

AND NOT (mice OR mouse).

**ComDisDom: 46.**

title((fragile X).

AND (Language OR communicat* OR conversation OR language OR linguistic* OR literacy OR literate OR narration OR non-verbal OR speak OR speech OR talk* OR verbal*)).

OR abstract((fragile X).

AND (Language OR communicat* OR conversation OR language OR linguistic* OR literacy OR literate OR narration OR non-verbal OR speak OR speech OR talk* OR verbal*)).

**CINAHL: 282.**

((MH “Fragile X Syndrome”)

OR TI “fragile x.”

OR AB “fragile x”).

AND (((MH “Communication”) OR (MH “Language”) OR (MH “Nonverbal Communication”) OR (MH “Verbal Behavior”)).

OR TI ((Language OR communicat* OR conversation OR language OR linguistic* OR literacy OR literate OR narration OR non-verbal OR speak OR speech OR talk* OR verbal*)).

OR AB ((Language OR communicat* OR conversation OR language OR linguistic* OR literacy OR literate OR narration OR non-verbal OR speak OR speech OR talk* OR verbal*))).

NOT ((MH “Mice”) OR (mice OR mouse)).

**PsycINFO: 583.**

(DE “Fragile X Syndrome.”

OR TI “Fragile X.”

OR AB “Fragile X”).

AND ((DE “Communication” OR DE “Nonverbal Communication” OR DE “Verbal Communication” OR DE “Language”).

OR TI ((Language OR communicat* OR conversation OR language OR linguistic* OR literacy OR literate OR narration OR non-verbal OR speak OR speech OR talk* OR verbal*)).

OR AB ((Language OR communicat* OR conversation OR language OR linguistic* OR literacy OR literate OR narration OR non-verbal OR speak OR speech OR talk* OR verbal*))).

NOT (mice OR mouse).

**Cochrane Database of Systematic Reviews: 5.**

**Cochrane Central Register of Controlled Trials: 44.**

(“fragile X”) AND (Language OR communicat* OR conversation OR language OR linguistic* OR literacy OR literate OR narration OR non-verbal OR speak OR speech OR talk* OR verbal*) in Record Title.

OR (“fragile X”) AND (Language OR communicat* OR conversation OR language OR linguistic* OR literacy OR literate OR narration OR non-verbal OR speak OR speech OR talk* OR verbal*) in Abstract.

- (Word variations have been searched).

**Google Scholar: top 35, sorted by relevance, citations and patents removed.**

(“fragile X”) AND (Language OR communication OR conversation OR language OR linguistic OR literacy OR literate OR narration OR non-verbal OR speak OR speech OR talk OR verbal) -mice.
